# Hierarchical motor control in mammals and machines

**DOI:** 10.1038/s41467-019-13239-6

**Published:** 2019-12-02

**Authors:** Josh Merel, Matthew Botvinick, Greg Wayne

**Affiliations:** DeepMind, London, UK

**Keywords:** Motor control, Computer science

## Abstract

Advances in artificial intelligence are stimulating interest in neuroscience. However, most attention is given to discrete tasks with simple action spaces, such as board games and classic video games. Less discussed in neuroscience are parallel advances in “synthetic motor control”. While motor neuroscience has recently focused on optimization of single, simple movements, AI has progressed to the generation of rich, diverse motor behaviors across multiple tasks, at humanoid scale. It is becoming clear that specific, well-motivated hierarchical design elements repeatedly arise when engineering these flexible control systems. We review these core principles of hierarchical control, relate them to hierarchy in the nervous system, and highlight research themes that we anticipate will be critical in solving challenges at this disciplinary intersection.

## Abstract (web summary)

Recent research in motor neuroscience has focused on optimal feedback control of single, simple tasks while robotics and AI are making progress towards flexible movement control in complex environments employing hierarchical control strategies. Here, the authors argue for a return to hierarchical models of motor control in neuroscience.

## Introduction

How neural circuits govern motor behavior has long been a central question for neuroscience research. In particular, it is a classical theme that the brain controls motor behavior through hierarchical anatomical structures. An early explicit proposal is owing to John Hughlings Jackson, who, by the 1870s, described the nervous system as a “sensorimotor machine”, consisting of a hierarchy of three evolutionary levels^1^. Since then, hierarchy both of anatomy and generation of behavior have been revisited in the study of instinct^2^, motivation^3,4^, and motor pattern generation^5,6^. Across these contexts, the focus has often been neuroethological, detailing the kinds of behaviors produced by species-specific nervous systems in their ecological niches. These ideas developed through study of the nervous system have inspired other disciplines, including robotics, with clear influence, for example, on the subsumption architecture^7,8^.

In recent decades, the theme of hierarchy has partially receded in motor neuroscience research, and the field has emphasized a largely complementary perspective, emphasizing task-specific optimality of movement^9^, with the contemporary version known as optimal feedback control (OFC)^10,11^. OFC is typically applied by postulating a cost function or formal definition of a task and asking what behavior is optimal with respect to that cost function. This perspective has been productive for motor neuroscience and facilitated the analysis of specific, well-defined motor behaviors. However, despite its great utility and its alignment with the experimental preference to study isolated behaviors in single tasks, the focus on specific movements runs contrary to the deeper interest in understanding the generation of diverse, ethological behaviors produced by nervous systems^12^.

OFC is a framework closely related to reinforcement learning (RL), which contemporary motor control for AI and robotics has widely adopted. We proceed by briefly reviewing computational approaches to motor control, focusing on the OFC framework, as well as reflecting upon recent developments in research involving control of complex, simulated physical bodies, including attempts to scale up OFC directly. However, as research into artificial control has developed, it has become clear that in addition to task objectives, system architecture design is also critical. OFC does not provide direct guidance on the design or interpretation of systems that must perform many behaviors or which reuse and compose overlapping skills to solve multiple tasks. We therefore formulate a set of core design principles of hierarchical systems in the context of motor control, which are synthesized from the AI research literature. In essence, recent work in AI has circled back to themes that were more central in earlier eras of neuroscience. This prompts us to take a fresh look at the neuroscience literature through a focused survey, which highlights how the core design principles help us make sense of hierarchical structure and function in the vertebrate nervous system. Both AI researchers engaging in the design of motor control systems and motor neuroscientists attempting to understand how specific nervous systems produce movement share many interests; we believe these fields will continue to benefit from interdisciplinary collaboration, so we close by highlighting some of these areas of overlap.

## Computational approaches to motor control

**Fig. 1:**
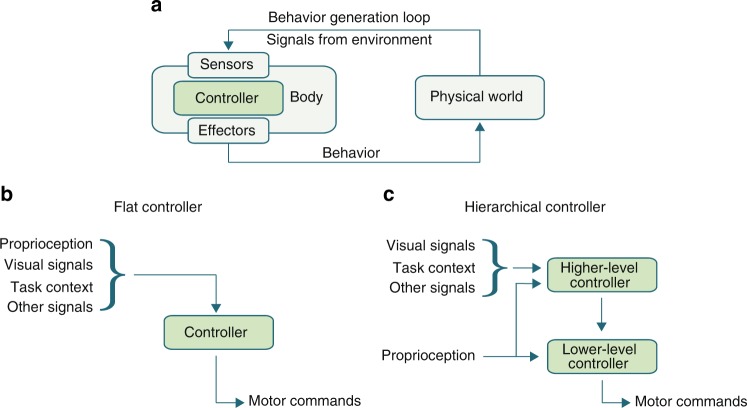
**a** Interaction cycle between an embodied control system and a physical environment to generate behavior. **b** A flat controller with no architectural segregation of different inputs. **c** A basic, brain-inspired two-stage hierarchy: a lower-level motor controller directly generates motor commands to the effectors based on input from proprioceptive sensors and modulatory input from a higher-level controller, which is responsive to additional signals, including vision and task context signals.

The challenge of motor control, both for animals and artificial systems, is to coordinate a body to produce patterns of adaptive movement behavior that satisfy objectives of the agent. When studying motor control with quantitative models, we consider a body in an environment, governed by a controller. The controller(or policy) receives observations from sensors, which measure features of the state of the system, and produces control signals that command the effectors. The controller runs in closed-loop with the body and environment, actuating the effectors based on online feedback from sensory observations to produce temporally extended behavior (Fig. 1a). For comparison, we depict a flat controller (Fig. 1b) as well as a minimal example of a hierarchical controller (Fig. 1c), in which high-level and low-level controllers receive different inputs and the motor commands are generated by the low-level controller with some input from the high-level controller.

Beyond the basic control system elements, specific control schemes may involve forward or inverse models^13^ (Here we focus on dynamics models. A distinct class of model supports coordinate transformations via forward and inverse *kinematic* models), and in biology, animals may use “internal” versions of these models^14,15^. Forward (dynamics) models predict the future state of the animal’s body and the environment given the current state and an action, either real or imagined. Internal forward models are used to predict the future consequences of actions. Comparing these predictions with sensory inputs enables filtering-based estimation of body and environment state. Forward models can also be used for action selection, as they allow an animal to “try out” actions using the model before acting with the real body. Inverse (dynamics) models form a special class of controller. They infer the action that takes the animal from the current state to a future outcome state. If this future outcome state is the “goal” of the animal, the inverse model generates the action that aims to achieve it.

OFC frames motor control as an optimization problem and was proposed as a normative theory of biological motor control^10^; this consolidated principles relatively well understood in movement neuroscience^16^. At present, OFC is the dominant framework used by motor neuroscientists to explain volitional control^17,18^. Earlier frameworks had recognized the value of optimizing movement trajectories^9^, but OFC emphasizes the importance of leveraging sensory feedback to produce task-optimal corrective responses to unexpected perturbations. As such, the key prediction that differentiated OFC from related proposals was that movements produced by animals correct for perturbations only to the extent needed to optimize the task. The OFC framework was generalized to encompass essentially all approaches that use closed-loop, feedback-based control, where the behavior generated is supposed to optimize a cost function (or goal)^11^. The broadened OFC framework consists of three principles: (1) Motor control is generated to optimize an objective function. (2) Deviations from an intended trajectory that arise should be corrected by leveraging sensory feedback in a task-optimal fashion. Together, these first two principles imply that online correction of movements should prioritize task-relevant dimensions (a “minimum intervention principle”). (3) Internal models help compensate for sensory delays and assist with state estimation.

From a contemporary perspective, the principles of OFC, including the utility of feedback and sensory delays, are widely accepted. The commitment in OFC that is perhaps most open to fundamental dispute is whether the controller really optimizes an objective (and what objective?). However, at its broadest, the OFC framework is fairly inclusive about what constitutes an objective. Efficient movement need not be a direct objective, but will indirectly emerge out of coordinating movement to rapidly solve tasks. So, if an animal is optimizing movement for solving a sequence of tasks, the efficiency of the movement is indirectly incentivized in order to facilitate the concrete task goals. Despite this theoretical generality, until recently is has not been widely feasible to consider task objectives more complex than those related to production of specific movements on short horizons.

## Motor control of synthetic systems

The optimization framework associated with OFC has been widely popularized in the context of “deep reinforcement learning” (Deep RL) (Deep RL refers to reinforcement learning that employs deep learning, or the use of deep neural networks.). The primary challenge of implementing optimal control approaches is generating the optimal control law (i.e., controller). For specific control problems described by known equations involving simple dynamics and cost functions, or problems formulated in low-dimensional state and action spaces, optimal controllers can be computed exactly. Specifically, one of the most fundamental and computationally straightforward ways to derive an optimal controller is through dynamic programming^19,20^. But for the control of more realistic, high-dimensional bodies, the design of the approximation scheme, learning algorithm, or numerical approach to produce the controller is important.

Specific, contemporary approaches often reformulate or restrict the generic problem in order to make it computationally tractable. A widespread algorithmic technique is to look for locally optimal control laws instead of globally optimal control laws. Examples of locally optimal algorithms include model predictive control^21^ or specialized planning methods^22,23^, which enable control of humanoid systems. However, planning approaches such as these are *model-based*, meaning they require access to the simulator within the planning computation; this is only available to an agent or animal if it possesses a high-quality forward model, possibly learned from previous experience. If there is no pre-existing or learned model of the environment, the alternative is to directly learn the policy (or, alternatively, a representation of the values of actions) via model-free RL^24^.

Over the last few years, there has been an explosion of interest in producing Deep RL agents that are trained in simulated environments. Progress made towards playing Atari games from images^25^ and navigating virtual environments^26^ have inspired considerable follow-up research. In parallel, there has also been significant effort applied towards control of articulated bodies in simulated physical environments^27^, with broad interest facilitated by the release of research environments^28,29^, which build accessible interfaces for underlying physics simulators such as MuJoCo^30^. These physics-based control (or continuous control) problems involve training a controller to produce an action-vector of continuous values, which actuate a physically simulated body, in order to optimize objectives in a task. Although primarily studied by Deep RL researchers for algorithm development, these challenges essentially amount to motor control. The approaches used in simulated environments also overlap with learning-based approaches for robotics research^31–34^. Of course, although significant development has occurred in recent years, many core ideas in Deep RL research were anticipated by earlier research^35^, including neural network control for graphically rich environments in the NeuroAnimator^36^, as well as design of impressive controllers for physically simulated humanoids^37–39^ and animals^40^.

Robust control of physically simulated humanoids, especially without access to the simulator for planning, is a challenge that has made progress in recent years. End-to-end learning approaches with relatively simple policy architectures (e.g., feedforward policies) are capable of producing simple locomotion behaviors^41^ and traversing obstacle courses^27^. In particular, Heess et al.^27^ pushed OFC to a certain extreme: motor behavior was generated via a simple feedback controller trained entirely end-to-end with deep RL to solve a single task, consisting of a distribution of more specific obstacle courses. The resulting policy was robust and responded well to random, procedural terrain variations as well as interactive perturbations by a human. In this work, the sensory observations consisted of feature-based height-maps of the terrain, similar to approaches in animation^42^. Subsequent work has since demonstrated the ability to solve similar problems from egocentric proprioceptive information and sensory information from touch sensors and egocentric cameras for a more ethologically plausible sensory embodiment^43^. Although sensors and effectors of simulated agents are not accurate models of those found in animals, it is nevertheless clear that simulated embodied agents face similar perceptual and motor challenges as real-world animals (or robots).

However, although end-to-end Deep RL approaches to motor control have expanded the scope of OFC, there are a number of difficulties. For settings with narrow objectives, such as running forwards, environment variations during training can induce robust behaviors. But for this to work, careful task design using a balanced curriculum is often needed^27^. And whereas intrinsic ethological drives of biological organisms are quite varied (including feeding, fighting or fleeing, and fornicating), typical Deep RL agents exist in a universe that consists of only a single, comparatively narrow objective. Broader challenges include dealing with changing objectives, learning behaviors that are reusable, and rapidly adapting to solve novel tasks. So, although there is clear value in scaling up OFC, it is far from the whole story of how animals generate motor behavior, and these broader challenges bring us back to aspects of motor control that were central in earlier work in both AI and neuroscience. To more efficiently solve complex control problems, many recent innovations relating to hierarchical system architecture are being developed. In the subsequent section, we will present core principles of hierarchical motor control. These principles reflect our distillation of older ideas, points that have been made in recently published work, as well as more ‘craft-level’ insights shared among researchers currently working in the field. For a concrete illustration of a simple, contemporary architecture reflecting versions of many of these principles, see Box 1.

Box 1 Reusable motor skills for hierarchical control of bodies

End-to-end RL with a “flat” controller initially explores the space of possible behaviors through uncoordinated, unstructured movements of each joint independently. For a complicated, humanoid body, intelligent behavior in this space is a needle in a haystack, making the search for task solutions a difficult problem. To promote a diversity of behavior as well as the exploration and discovery of new ones, the neural probabilistic motor primitives (NPMP) architecture has been introduced^44^, which expresses a set of robust, human-like motor behaviors as a basis for further task learning. The system is first trained using motion capture data of humans performing movements. The motion capture data are time series of configurations of the body and joints. The details of the construction of the system are not critical, but, to give some insight, for each motion capture snippet, a neural network is trained by RL to produce actions, *a*_*t*_, such that the resulting movement trajectory approximately tracks the kinematic position of the body in the original reference motion. Then, these movement controllers are combined or “distilled” into one large model that can track any of the movements given a description of the near future path of the body, x*_t_. A coding space, *z*_*t*_, in the system comes to represent each of these movements and allows interpolation among them. Downstream of the code is a motor policy, which, when cued with *z*_*t*_ and proprioceptive information *s*_*t*_, is able to generate patterns of human-like movement autonomously. Thus, exploration of the space of human-like movements becomes possible by varying the input *z*_*t*_ to the motor policy. To this low-level motor system, a high-level controller can be attached to solve complicated tasks in virtual environments. The high-level controller has full visual input and is provided task information, *o*_*t*_. It learns by RL to produce actions of the same size as the coding space, which modulate the movements carried out by the low-level policy. The NPMP's modular, hierarchical design has made it possible to solve complicated problems otherwise of great difficulty for flat RL. See supplementary materials (videos and associated captions) for examples of motor reuse.

## Core principles of hierarchical motor control

Researchers engaged in the study of hierarchical control believe that hierarchy can add value for issues ranging from effective exploration and planning to transfer and composition of skills. Synthesizing the literature, we have attempted to clarify and summarize core principles of hierarchical control that we believe facilitate design and interpretation of hierarchical systems. In particular, the principles we identified are well motivated when considering systems capable of generating a wide range of motor behaviors across multiple settings. The principles are elaborated below and a brief description and motivation for each principle is summarized in Table 1.

**Table 1:** Summary of key principles of hierarchical control.

Core principle	Brief summary	Motivation/utility
Information factorization	Different information is routed to different subsystems.	Factored learning can require less experience per subsystem. Subsystems are invariant to hidden information and therefore are reusable across contexts.
Partial autonomy	Lower-level systems function somewhat autonomously, with modulation from higher-level systems.	System is more robust and lower-level does not require costly micromanagement.
Amortized control	Movements that have been successfully executed multiple times are compressed into a system that can rapidly reproduce them.	Re-execution of frequently repeated movements should be more computationally efficient than novel variations.
Modular objectives	Specific subsystems may be trained to optimize specific objectives, distinct from the global task objective.	Training of subsystems can leverage error signals that are denser or more well known than the global task objective.
Multi-joint coordination	Movement is produced in a manner that reflects common patterns across the body.	Exploration and action-selection can exploit commonly co-occurring multi-joint patterns.
Temporal abstraction	Common temporal motifs are abstracted.	Behavior specification or planning can occur at a coarser timescale.

### Information factorization

Information factorization refers to the property of hierarchical systems that involves providing partial or pre-processed information to certain parts of a system (c.f. information hiding^45,46^). In our simple example (Fig. 1), this principle is illustrated by different sensory signals being routed to the high- and low-level controllers, respectively. Although a flat policy could, in principle, integrate all available information and produce controls directly, a system with fewer inputs per module is likely to learn more efficiently. Furthermore, by segregating information immediately relevant to the low-level controller from information that only needs to modulate the low-level controller in a low-bandwidth fashion (e.g., via an inter-layer bottleneck), the low-level controller is likely to generalize better. By construction, the information routed to it is invariant to many possible contexts, and it only directly processes the subset of sensory information that the behavior it is responsible for generating depends upon. Concretely in the example in Fig. 1, the higher-level controller might provide modulatory signals as simple as steering signals, whereas the low-level controller may have to produce high-dimensional locomotion motor patterns.

This idea is connected to a view of reinforcement learning in which subsystems that have access to different information are able to share appropriately abstract behavior across contexts^47,48^. For example, while visually guided locomotion in the context of a particular task may involve focusing on specific elements in the visual scene that do not transfer entirely to a new task, the locomotor movement patterns may generalize. In this example, low-level behavior is more invariant owing to information factorization. However, it can also be the case that high-level behavior is invariant. Sufficiently abstract goals or intentions permit many distinct low-level movements to achieve them, so a high-level controller with limited access to body state may communicate an abstract goal that does not fully specify the required details of the movement, leaving it to the lower-levels to sort out the details. That some goals or tasks can be solved by a multiplicity of execution details (“motor equivalence”) has long been recognized as important in movement science^49,50^ and has also been identified as relevant for robot control^51^.

### Partial autonomy

Partial autonomy refers to the property of certain types of hierarchical systems that the lower-levels of the hierarchy can semi-autonomously produce behavior even without input from higher-levels. This principle is related to the intuition underlying the subsumption architecture^7^: build low-level controllers that function autonomously; then add modulatory control layers such that the overall system can produce more behaviors. The insight reflected in this approach is that robustness can be achieved if lower-layer controllers are sufficiently autonomous (albeit for a more limited range of behavior), such that removal of the higher layers leaves the lower-layer generated behavior intact. This style of architecture is evocative of the brain^8^, insofar as for many animals, considerable functionality remains in animals with substantial portions of the central nervous system removed, as we discuss later.

This partial autonomy is related to information factorization insofar as a lower-level system should have adequate information to be partially autonomous. For example, a low-level locomotion controller may simply produce straight-ahead (or randomly-directed) walking behavior in the absence of inputs from the higher-level controller, but this locomotion can still be stabilized by proprioceptive feedback. Partial autonomy also pertains to a class of robustness having to do with appropriate responsiveness to perturbations. Consider a setting in which an agent (or animal) is engaged in a behavior (e.g., walking) and, owing to something unanticipated in the environment, the agent slips or is perturbed. Although “default” behavior may be somewhat automatic, a role for higher-layers might be to detect that something unexpected has occurred via monitoring what is unfolding, and respond with the appropriate modulation of the overall behavior. So, whereas simple walking may be performed adequately by lower-levels of control, increasingly intelligent responsiveness may require rich sensory information as well as the ability to assess the environment for safe affordances (e.g., something to hold onto in response to slipping).

### Amortized control

In order to accelerate computation of behaviors that require complex motor coordination, hierarchical systems can benefit from amortized control. Amortized control refers to a wide range of approaches that involve training a lower-level system to produce appropriate behaviors for a behavioral context or modulatory signal, without having to engage in a costly process. For example, although it is quite costly to plan or optimize movements entirely from scratch, once movements have been produced, it should be possible to train a “reactive” subsystem that can reproduce these movements repeatedly without redundant planning. This principle is related to partial autonomy, as it may involve the production of a semi-autonomous subsystem, but the emphasis of this principle is on the benefit with respect to computation attained through caching previously obtained solutions.

Motivated by this insight, it has been demonstrated that policies produced via trajectory optimization could be distilled into a neural network that could then be reused interactively^52,53^. Similar ideas have also been explored^44,52–54^, reflecting a shared intuition that well-behaved trajectories obtained from various sources can be used to train a neural network that may generalize from the examples. From a system perspective, this is a kind of self-supervised learning where trajectories generated by one (presumably slow or costly) mechanism are used to train another part of the system to produce equivalent behavior in an amortized fashion.

### Modular objectives

Many examples of neural networks applied to control problems use “end-to-end” optimization^25^; that is, there is a single task objective, and the entirety of the architecture maximizes this singular objective. However, the broad alternative is that control systems have some functional separation of roles by subsystem, and different modules benefit from being trained by distinct modular objectives. A specific, practical, and popular approach trains a controller to solve a task while also training a set of internal representations to predict future sensory data^26,55,56^. This approach to learning internal state representations can improve experience efficiency by leveraging dense self-supervised objectives to train perceptual and memory modules, whereas task reward can still provide learning signals for the controller. This approach is “heterarchical” insofar as different objective functions, consisting of a predictive objective as well as a policy improvement objective, are imposed in parallel on different parts of the overall network architecture.

Another classic approach involves the overall system specifying subordinate objectives for modular subsystems, while maintaining the priority of a high-level objective. Paradigmatically for control problems, a high-level controller can communicate a goal to a low-level controller, which serves both as instruction to modulate low-level behavior and also as a reference for learning. Such an approach amounts to a divide-and-conquer strategy^57^, and has been implemented via reinforcement learning^45^. For example, in locomotion control, a high-level controller may decide to move in a certain direction, provide a signal to the low-level controller as instruction, and this signal also serves as a dense teaching signal that the low-level controller learns from as it assesses how well it stays on the instructed course. In such schemes, the low-level controller is trained to satisfy its received instruction, whereas the high-level controller intelligently programs these objectives to solve a more global task. Most work on this idea has used fixed forms of the cost function for the low-level controller^58,59^, but other work has explored how to learn more abstract goal spaces^60^.

### Multi-joint coordination

Although it may make sense to be able to modulate or directly control single muscles or joints in specific contexts, most control is perhaps better thought of as selective activation of established motor synergies. There are many variations on the motor synergy concept^61^; here we mean functional couplings of different joints or muscles such that motor control operates at the level of multi-joint coordination patterns rather than through independent control of all joints. Producing actions at this slightly higher level of abstraction can facilitate exploration and learning of new skills as well as simplify planning. This is perhaps most readily apparent in a setting like reaching and grasping, where random movement of all degrees of freedom independently will be ineffective, but random movements in the subspace of hand configurations encountered during grasping will lead to more effective interactions.

Perhaps, the conceptually most straightforward way to implement multi-joint coordination is to perform control or planning in a pre-specified, low-dimensional space. For well understood classes of movement, such as locomotion, versions of low-dimensional control have been around for a while, such as specifying the walking in terms of a simplified body model and computing leg movements to achieve the target movement of the center-of-mass^62^. This strategy has been advocated more generally^63^, and a relatively recent representative performs low-dimensional planning for locomotion in a hand-designed space that interacts with a low-level controller^64^. An alternative to hand-engineering the low-dimensional control space involves unsupervised learning (or self-supervised learning) of sensorimotor primitives in order to produce a learned low-level controller^11,65^.

### Temporal abstraction

Temporal abstraction simplifies the specification of behavior that endures over extended time intervals via higher-level controllers operating at a coarser temporal resolution. For example, in the context of locomotion, a higher-level controller may instruct a low-level controller at a less-frequent timescale on where to navigate (or when to turn), but the actual movement is executed over an extended duration by a lower-level controller that operates at the full temporal precision required for motor behavior. Through this scheme, a trade-off is established, whereby the high-level controller may cede control precision, but gain in time-horizon through the reduced temporal resolution—this enables the high-level controller to more easily discover or plan behavior that endures on a longer natural timescale.

In the hierarchical reinforcement learning literature, a number of schemes have been proposed that focus on leveraging temporal abstraction^66^. In particular, the options framework, which involves high-level transfer of control to self-terminating subroutines, has been highly influential^67^. Deep RL also can incorporate temporal abstraction^68^. The conventional focus on temporal abstraction as opposed to multi-joint coordination in hierarchical RL makes sense when one appreciates that many canonical RL problems have comparatively low-dimensional, discrete action spaces. In settings where control is simple, the only way to abstract control complexity is in the time domain. For problems with high-dimensional continuous action spaces such as control of bodies or robotic manipulators, multi-joint coordination can be more critical than temporal abstraction^63^. But of course, longer-term motor planning and behavior selection do require temporal abstraction.

Temporal abstraction can also be implemented via commitment to a task, goal, or context. That is, agents may, for a period of time, select a behavioral mode or “goal” and all behavior executed could be directed in support of this goal (this overlaps with the use of goals for modular objectives, but is distinct in motivation). In such an implementation, the selected goal is a form of high-level action and allows for coarser control, both temporally and in terms of level of precision of the goal state. Whereas “state abstraction” with respect to goals is distinct from temporal abstraction, the two are correlated in many settings—for example, in navigation settings spatially distal goals are usually temporally distal as well^45^.

## Neurobiological hierarchical motor control

As noted earlier, the renewed relevance of hierarchy in AI returns attention to a theme that was central not only in earlier AI research, but also in earlier neuroscience research. With this in mind, we turn now to our survey of hierarchy as relevant in neuroscience research on motor control, considering how the principles described in the previous section relate to known properties of brain function. The nervous system of higher vertebrates controls movement through a distributed set of structures that are both anatomically and functionally hierarchical (see Box 2 for overview). Of course, in very broad terms, that the nervous system is hierarchically structured is something that is widely accepted and touted at the level of introductory textbooks. But more specifically, as there are distinct ways for a system to be hierarchical, we believe the principles of hierarchical control emerging through the study of artificial systems help us make sense of even the detailed elements of the biological motor control system.

Our brief survey will primarily focus on the functional role of key parts of the nervous system in the context of motor control. Historically, this has been investigated through now classic studies involving the removal of portions of the brain, as well as neural recording and stimulation. This classic literature is bolstered by relatively more recent work that considers loss of function in the context of inactivation and removal specifically of motor areas. The review will proceed from lower-level motor structures up to “higher” brain regions, and we will emphasize the relevant principles introduced in the previous section where appropriate.

Box 2 Review of the neuroanatomical hierarchy

The diagram depicts an abstraction of the hierarchical anatomy of the mammalian nervous system. The scheme is, insofar as possible, a consensus view of previous hierarchical interpretations^3,4,6,69^, with the intent of serving as an uncontroversial foundation. A natural entry point is the motivation regulation nuclei. The central nervous system receives information about the body via signals from the gut, level of hydration, hormones, blood sugar levels, and other measures. Much of this information arrives via structures such as the hypothalamus, which then communicates information related to motivational state to other parts of the brain. These signals related to basic drives (hunger, arousal, etc.) directly or indirectly will guide behavior. Subcortical structures, such as the basal ganglia, are responsible for regulating behavioral context and modulate the activity of more foundational motor generators in the brainstem and spine, which also receive limited sensory information via subcortical sensory structures. In parallel, motivational (“drive”) information and sensory information are processed in cortical areas which in turn modulate behavioral context and ultimately allow for the use of more processed information to inform motor coordination via motor cortical areas.

A common motif across specific hierarchical models that have been proposed is the presence of multiple routes of information transmission and motor coordination. In terms of sensory input, dual sensory input pathways transmit information along a subcortical pathway as well as a cortical pathway^4^. Similarly, there are direct subcortical pathways from motivational centers (or what has been referred to as the limbic system) to brainstem nuclei that activate motor patterns, as well as indirect routes, either via the basal ganglia or through frontal cortices^3^. This multi-pathway motif structurally reflects some of hierarchical control principles, with multiple layers to the system being partially autonomous, each having access to partial and differently processed information.

### “Lower-level” movement centers

It is an incredible feature of the nervous system that substantial parts of the brain can be removed while preserving significant functionality. This broadly reflects the relevance of the hierarchical control principles of partial autonomy as well as information factorization—brain subsystems receive relevant partial information and can control some movement even without higher-level inputs. The spine, even in spinalized preparations, is responsive to somatic sensory feedback and can act semi-autonomously from the brain to coordinate multiple joints over time. Spinal circuits are capable of both generating their own spatiotemporal coordination patterns, such as “fictive” locomotion^70^ via central pattern generators (CPGs) as well as modulating activity locally via sensory reafference^71,72^. There is also a rich literature on spinally controlled time-varying movement primitives involving coordination of multiple joints to control to an end-point or to trace a “virtual trajectory”^73–75^. While difficult to assess directly, it is believed that these primitive spinally generated movements and patterns are relevant for humans^76^, with the basic movements that support walking behavior having an innate component that arises early in development^76,77^.

At the level of the brainstem, much of our knowledge comes from experiments involving decerebration as well as stimulation. We know a great deal about the functional anatomy of decorticate and decerebrate cats^78^. Depending on precisely where decerebration is performed, animals retain the ability to walk spontaneously, or only under stimulation of nuclei such as the mesencephalic locomotor region (MLR). In intact animals, nuclei such as MLR receive inputs from relatively higher regions including the hypothalamus and basal ganglia that modulate locomotor behaviors. Locomotor nuclei do more than generate oscillatory patterns—some version of which is already handled by the spine. Instead, these nuclei orchestrate slightly more abstract multi-joint coordination of movement patterns and regulate locomotion. They also incorporate cerebellum-derived signals, somatic feedback, and inputs from other sensory systemts to help coordinate movement.

### Subcortical “mid-level” movement regulation

Where decerebration removes the entire cerebrum, decortication refers to the removal of cortex without damage to thalamus or basal ganglia, so essentially all subcortical structures are intact, modulo atrophy owing to removal of significant sources of inputs. Cats and dogs with their entire cortex removed often generate superficially normal behavior after a recovery period^78^. In an early review into the behavior of decorticate cats, David McK. Rioch vividly observed: “During the first few days following the operation, when the animal walks into a corner, it continues to push forward, butting its head against the wall. Struggling, sprinting, and climbing reactions may occur, but escape from the corner is accidental. Later on the animal will turn aside from an obstruction after having bumped into it, or after having merely touched it with its whiskers or ears”^79^.

This description of the behavior of decorticate cats reveals a number of critical features from the perspective of hierarchical control: (1) cortex is not required for a significant amount of the behavior generated by the cat. This reflects partial autonomy as well as amortized control, insofar, as stereotyped movements are “habitual”. In particular, we also know that decorticate animals with intact basal ganglia can initiate goal-directed locomotor behavior^80^. The basal ganglia then appropriately modulates the brainstem locomotor nuclei, which in turn modulate spinal CPGs. (2) Subcortical structures can select among different modes of coordinated behavior, possibly reflecting short-term temporal abstraction and multi-joint coordination. Specifically, it has been proposed that motor program selection is performed by the basal ganglia, normally informed by inputs from cortex and thalamus^6^. This is also consistent with recent work correlating neural activity in striatum with moment-to-moment sequencing of movement “syllables”^81^. (3) While sensory-guided insight is impaired upon removal of cortex, residual sensory information that has been processed through non-cortical pathways remains available, reflecting appropriate information factorization. (4) Certain forms of learning still occur, obviously mediated via non-cortical circuitry^79,82^. It is believed that learning of motor coordination is mediated by cerebellum and learning related to action selection is mediated by basal ganglia^83,84^. This is consistent with the broader literature on the basal ganglia being involved in the learning and deployment of context-triggered habitual actions, with this circuitry thought to implement something like reinforcement learning^85,86^.

Further, complex patterns of behavior associated with motivational states are also substantially intact in decorticate animals. For example, decorticate male rodents are even capable of generating the complex motor repertoire required to engage in copulatory activity and sire pups^87^. A fully integrative perspective should aim to include drive assessment and selection of motivational-behavioral contexts as part of the hierarchical control system. In particular, the hypothalamus is involved in regulating motivational state, and stimulation of hypothalamic sites produces the motivation to engage in certain behaviors^88,89^. Contemporary research continues to corroborate the perspective that evoked behaviors mediated by discrete hypothalamic regions reflect specific goals or motivated states^90^, with certain hypothalamic nuclei more specifically implicated in aggressive responses^91^ as well as sexual behaviors^92^. Our inclusion of drive regulation as part of hierarchical control connects with historical characterizations of hypothalamus as related to movement regulation^93^ or hierarchical interpretations that place hypothalamus atop the motor control hierarchy^4^. These motivated states signal to other areas to initiate behaviors suited to the satisfaction of the motivated state. And consistent with partial autonomy and the structured information factorization in the nervous system, there seems to be a direct motivation-driven subcortical system that handles coarse behavioral selection, as well as a secondary pathway that is frontally mediated and refines motor objectives or goals on a longer horizon^3^.

### Cortical “high-level” control of movement

Despite the fact that many decorticate mammals show superficially normal behavior, clear deficits become apparent upon closer inspection, and these deficits are more dramatic in primates. This was initially a source of confusion for David Ferrier and Friedrich Goltz in the late 19th century. Although Goltz and others could produce non-primate decorticates that showed the kinds of behavior described in the preceding sections, Ferrier found significant impairments amounting to partial paralysis when only motor cortex was removed in a monkey^94^. Convergent evidence comes from humans in clinical cases involving focal motor cortical damage owing to injury; strokes have a substantial affect, resulting in transient partial paralysis, followed by considerable recovery, though without recovery of fine motor skills^94^. Although there is still uncertainty about the role of motor cortex^95^, at least as early as Bernstein, it has been appreciated that increasingly sophisticated organisms need elaborated, higher-level motor structures to solve general motor challenges; these elaborations enable the generation of a broader repertoire of diverse motor responses and support the performance of extemporaneous, unrehearsed movements^5^. This flexible higher-level functionality or motor “wit” is what Bernstein termed “dexterity” and defined as: “finding a motor solution for any situation and in any condition”^96^. To facilitate this high-level function, Bernstein observed that higher-level structures are well integrated with telereceptors (i.e., “long-range” sensors that detect olfactory, visual, and auditory signals); on the basis of evolutionary and anatomical evidence, Bernstein argued that this factorized sensory stream informs high-level structures that coordinate or override stereotyped and automatic movements generated by lower-level structures^5,96^.

The settings in which higher-level structures are most relevant depend upon the specific behaviors for which the animal is adapted. For example, dogs and cats do not execute dexterous finger movements, whereas non-human primates, humans, and even rodents do^97^. And increasingly for animals that reach and exhibit dexterous finger control, direct cortical control of upper-limb extremities allows closer integration of visual and tactile information for hand-eye (and finger) coordination. To support sensory-guided fine motor control, which is required for dexterous manipulation, non-human primates and humans have more substantial direct projections from cortex to spine^80,98^. The anatomical variation continues even among primates, with fine motor control by humans even surpassing other primates^99^. More broadly, the general role for high-level structures in mediating sensory-rich control may be relevant in other niches; for example, legged traversal of precarious terrains, as performed by a mountain goat navigating small footholds, is also obviously dependent upon visual guidance for foot placement.

Recent studies involving targeted inactivation or removal of motor cortex provide evidence that supports this view that cortex refines movement, primarily in contexts involving precise sensory-guided control or dynamic motor improvisation. In rodents, the production of grasping behaviors has been localized to the rostral forelimb area (RFA), and long-duration intracortical microstimulation can generate reaching and grasping behaviors^100^ (paralleling similar results in monkeys^101^). Experimenters have demonstrated that transient, reversible, and specific deficits in pellet-grasping ability are produced in behaving rats when RFA is silenced via cooling^102^. In other experiments, rodents traversed a simple “obstacle course” with infrequent dynamic perturbations^94^. Although rodents with bilateral motor cortical lesions showed no significant deficits in navigating stable terrains, in the presence of dynamic perturbations, lesioned animals were unable to rapidly adapt their movements. The sensory-guided element of motor cortical control was perhaps most directly tested in experiments making use of a virtual environment that allows for the experimental dissociation of motor control and sensory feedback—researchers found that in response to experimental perturbations of the visual environment, the local cortical microcircuit in motor cortex was involved in producing corrective motor responses to situations where the actual sensory consequences did not match predictions^103^. Taken together, motor cortex appears required for fine-scale, dexterous motor control, especially involving sensory guidance, but motor cortex may not be required for stereotyped (autonomous and amortized) movements, consistent with previous interpretations^94,103^.

In yet other experiments involving rodents, complex, but non-dexterous, stereotyped motor trajectories that an animal learned in order to solve a task were preserved when motor cortex was bilaterally removed^104^. However, learning was shown to be dependent on the presence of motor cortex, which is interpreted as evidence for initial production of the movement being mediated by cortex, followed by tutoring of subcortical regions^104^, seemingly implementing a form of amortized control. However, the science of where amortized motor representations are stored (c.f. “automaticity”) remains unsettled as other findings suggest cortex may store certain learned patterns after being driven by exploration generated subcortically^105^.

The alternative to control being amortized, regardless of the neural locus, is that every movement is planned from scratch each time any movement is executed. It has been argued that planning or optimization occur via preparatory activity preceding movement, both for reaching behavior^106–108^ and in the context of decision-making tasks^109–111^. Although it remains an open question how the nervous system balances pre-movement planning with amortized control in ethological settings, we expect planning to be most beneficial for control of idiosyncratic movements or in settings in which control must be precisely micro-managed by sensory feedback. Insofar, as experiments which study preparatory activity employ paradigms in which animals engage in highly stereotyped behavior, it is difficult to know how to relate preparatory processes in these settings to ethologically relevant motor planning.

Two of the principles of hierarchical control that have not featured as prominently in this short review, despite being important for cortical function, are learning by modular objectives and temporal abstraction. It is beyond the present scope to review how the nervous system learns to extract structured information from sensory signals or encodes memories—these processes undoubtedly are governed by diverse learning signals (i.e., modular objectives). We also will not cover the various frontal structures that are even “higher” than the motor cortices. These structures are involved in planning and reasoning processes, which may result in the specification of goals; temporal abstraction certainly features prominently^112,113^.

## Shared challenges for biological and synthetic motor control

As the preceding section articulates, many of the interest areas pursued in recent AI work on hierarchical motor control find corresponding relevance in neuroscience. This makes evident a current opportunity for synergistic exchange between the two fields. We also emphasize that hierarchical control in AI is far from solved—despite significant progress in artificial intelligence research over the past years, there remain meaningful challenges in dealing with rich sensation, a broader range of tasks, rapid adaptation or improvisation, as well as object interaction and tool use. However, we are optimistic that we can make progress on these outstanding challenges. Towards this end, we highlight research themes that already have active interest, but which we believe deserve further attention.

### Towards full-scale body control

Theories of biological motor control must actually confront the problem of controlling a full-scale body in an environment for a range of tasks—we should aim to build models that both reflect the nervous system and function as controllers. For single-behaviors, motor control in simulation has already afforded a constructive setting in which to define biologically informed models, and various interesting research has been undertaken towards control of bodies, often with an emphasis on biomechanics and muscle-level control^114^. Previous efforts have generally considered control of certain movement behaviors, such swimming in lamprey^115^, control of locomotion in cats^116^ or humans^117^, as well as swimming and walking in salamander^118^. Efforts by Delp and colleagues have pushed to model biomechanical control of musculotendon-driven models^119^, including tendon-driven simulations of upper^120^ and lower limbs^121^; these models can be used to analyze specific movements and prepare surgical interventions. Despite the aforementioned efforts, which begin to demonstrate the utility of physics-based simulation for studying neural control, building controllers that capture meaningful diversity of behavior is a tremendous opportunity that remains, at present, underexplored.

To produce controllers that capture the rich behavioral diversity of biological organisms, two broad approaches are possible—train the system to solve diverse tasks or produce data-driven generative models of observed behavior. With task modeling, we acknowledge that real animals can solve a wide range of tasks efficiently, and we produce diverse behavior through defining tasks and learning algorithms. Intriguing forays have been made within neuroscience at handling multiple cognitive tasks^122,123^, albeit with the role of motor control quite restricted. The complementary approach is to produce data-driven generative models of animal behavior; specifically, this involves control of a physically simulated body in an environment with an aim of matching empirically observed reference behavior. As highlighted previously in this review, there has been some research into hierarchical control schemes for which animal or human motion capture is leveraged to produce a low-level movement controller^40,42–44,124–126^. A related idea that is more familiar within neuroscience involves building descriptive models of the behavior of an animal^127–129^, but fewer efforts have so far aimed to combine descriptive models of animal behavior with physically realistic control of movement.

### The structure of inter-region communication

At present, we do not fully understand what coding schemes brain regions use to communicate, and we are similarly uncertain how to specify information flow in synthetic hierarchical motor control systems. The default scheme for communication between layers or modules of learning systems is for the output of one layer to serve as an input to another layer. However, there are still various open questions—for example, should communication follow prescribed semantics? Learning systems will not necessarily result in interpretable inter-layer communication, unless structure emerges through the learning process or is encouraged explicitly. A second question is how, mechanistically, the outputs of one system should modulate another—whether activations from one layer should serve as simple inputs or if they should nonlinearly modulate their target, such as via multiplicative gating (e.g., see the “Transformer”^130^ or FiLM layer^131^). Yet another question concerns the level of resolution of the signals sent between regions—what is the balance between communicating abstract goals that only partially specify behavior versus communicating rich instructions that precisely tell the lower-level system what to do? Too intense micromanagement makes the function of a low-level system redundant, yet in certain cases it may be useful for a high-level system to entirely override low-level behavior.

To ground these issues in neuroscience, we can consider a specific debate in the field—Friston^132^ identifies a key difference between classes of proposed hierarchies as having to do with the semantics of signals sent from higher-level controllers to lower-level controllers, noting that “In active inference, descending signals are in themselves predictions of sensory consequences.” As an alternative, Todorov et al.^63^ advocated for the interface between the higher-level and lower-level controllers to be engineered and reflect insight into an appropriate set of variables well suited to the range of behavior. Although it is not yet clear which of these proposals, if either, corresponds to biology, the general point is clear—hierarchical systems must employ a language or code at the interface between layers or regions. Here, we do not propose to resolve this issue, but instead suggest that this area presents an opportunity for neuroscience and AI efforts to collaborate in proposing communication schemes and evaluating which are effective.

### Ethological motor learning and imitation

Animals and humans efficiently learn motor behaviors throughout life via active exploration, imitation of conspecifics, and subsequent refinement of skills. Although birdsong is a narrow behavior relative to primate motor control, it serves to illustrate some of the multiple requirements—evolutionarily initialized motor variability (“babbling”) in juvenile songbirds is shaped into skilled behavior by a process of vocal imitation learning followed by self-directed rehearsal^133–135^. More broadly and across species, intrinsically motivated active exploration is required to learn both about the environment as well as how self-generated behavior can affect the environment^136^. In humans, imitation-based learning begins with observing the movements of others, but can involve inference of the goals of the demonstrator as well as intelligent exploration to imitate their movements or goal-directed activity^137^. Further, it is thought that non-verbal pedagogical behavior is an evolutionary adaptation^138^, and related imitative behavior may have antecedents in the gestural communication already present in some other species^139^.

At present, the conventional forms of artificial “imitation learning” do not yet match the biological inspiration. Contemporary approaches require that demonstrations are essentially performed on the body of the student (e.g., via teleoperation), granting first-person access to demonstrated behavior. Learning from this information is referred to as behavioral cloning^140^, and usually is implemented as a regression from demonstrated states to actions^141,142^. But recent advances take steps toward more natural imitation. For example, adversarial imitation^143^ can scale to humanoids even without access to actions^124^, possibly from only allocentric, video demonstrations^144^. Another particularly exciting and naturalistic development is “one-shot imitation learning”, where, after training, the system is presented with a novel demonstration and immediately attempts to reproduce that demonstrated behavior^145^; this style of approach has also been employed for humanoids^44,146^. As an intermediate representation that supports one-shot observation and imitation of demonstrations, systems may possess an embedding space that simultaneously encodes the demonstrated behavior and reflects what the agent will do. Conceptually, this is similar to the representation identified for mirror neurons^147^.

## Concluding remarks

In this review, we have attempted to reflect upon the principles of motor control in biological nervous systems as well as ideas for designing motor control architectures for synthetic systems. Both neuroscience and artificial intelligence research have clearly benefited from taking the perspective that behavior should be optimized to solve tasks. But overemphasis on isolated, straightforward motor control tasks obscures meaningful challenges. Recent work in AI involving efforts to scale motor control to richer and more diverse behaviors, has catalyzed a shift in focus towards hierarchical systems capable of handling a diversity of tasks. This trend points to themes that were central in earlier eras of both artificial intelligence and neurobiological motor control research. Moving forward, we propose that effort should be focused on building models that can generate the flexibility and breadth of motor behavior produced by animals. Once embraced, this perspective will accelerate efforts to reverse engineer the motor system.

## Supplementary information

Description of Additional Supplementary Files

Supplementary Movie 1

Supplementary Movie 2
