# Supplementary material for: Hierarchical motor control in mammals and machines
Source: Nat Commun. 2019 Dec 2;10:5489. doi: 10.1038/s41467-019-13239-6 (PMC6889345; doi:10.1038/s41467-019-13239-6)
Supplement: Supplementary file 1 — Description of Additional Supplementary Files [file 41467_2019_13239_MOESM1_ESM.pdf]

Description of Additional Supplementary Files

File Name: Supplementary Movie 1
Description: **NPMP reuse for Forage task.** Example of low-level controller from neural probabilistic motor primitives architecture being reused by training a high-level controller to perform a foraging task in a maze. This example maze is randomly sampled from a procedural maze generator. The high-level controller receives visual information, which is hidden from the low-level controller, and the visual information is required to solve the task.

File Name: Supplementary Movie 2
Description: **NPMP reuse for Gaps task.** Example of low-level controller from neural probabilistic motor primitives architecture being reused by training a high-level controller to run along a corridor with gaps. Note that the high-level controller was trained to perform run along corridors with varying gap size. Depicted here is a probe evaluation with gaps at roughly the limit of what the controller was able to manage. Examples of failures are included. The high-level controller receives visual information, which is hidden from the low-level controller, and the visual information is required to solve the task.
